# Effectiveness of particulate matter filtration: systematic review and meta-analysis

**DOI:** 10.1590/0034-7167-2024-0454

**Published:** 2026-03-16

**Authors:** Francieli Faustino, Helenize Ferreira Lima Leachi, Aline Franco da Rocha, Ana Paula Silva, Rosângela Marion da Silva, Renata Perfeito Ribeiro

**Affiliations:** I Universidade Estadual de Londrina, Londrina, Paraná, Brazil; II Universidade Federal de Santa Maria. Santa Maria, Rio Grande do Sul, Brazil

**Keywords:** Filters, Adsorption, Chemical Compounds, Effectiveness, Organic Compounds., Filtros, Adsorción, Compuestos Químicos, Eficacia, Compuestos Orgánicos.

## Abstract

**Objectives:**

to assess the effectiveness of filters in filtering particulate matter.

**Methods:**

a systematic literature review was conducted with the research question: which filters efficiently adsorb chemical and/or biological particles present in the air when tested in scientific interventions? The databases used were Medical Literature Analysis and Retrieval System Online, Web of Science, Scopus, Latin American and Caribbean Literature in Health Sciences, American Chemical Society, Royal Society Chemistry, Sci Finder, and Scientific Electronic Library Online.

**Results:**

forty-seven studies were included in this review. The High Efficiency Particulate Arrestance filter was tested in 36 studies. The seven studies used for the meta-analysis were homogeneous.

**Conclusions:**

filtration performed using the High Efficiency Particulate Arrestance filter reduced particulate matter concentrations by 70% to 80% in the assessments and interventions presented, contributing to the evidence that this filter reduces particulate matter concentrations.

## RESUMEN

**Objetivos:**

evaluar la eficacia de los filtros para filtrar material particulado.

**Métodos:**

se realizó una revisión sistemática de la literatura con la pregunta de investigación: ¿Qué filtros adsorben eficientemente partículas químicas y/o biológicas presentes en el aire al ser probados en intervenciones científicas? Las bases de datos utilizadas fueron *Medical Literature Analysis and Retrieval System Online, Web of Science*, Scopus, Literatura Latinoamericana y del Caribe en Ciencias de la Salud, *American Chemical Society, Royal Society Chemistry, Sci Finder y Scientific Electronic Library Online*.

**Resultados:**

se incluyeron 47 estudios en esta revisión. El filtro de alta eficiencia para la retención de partículas se probó en 36 estudios. Los siete estudios utilizados para el metanálisis fueron homogéneos.

**Conclusiones:**

la filtración con el filtro de alta eficiencia para la retención de partículas redujo las concentraciones de material particulado entre un 70% y un 80% en las evaluaciones e intervenciones presentadas, lo que refuerza la evidencia de que este filtro reduce las concentraciones de material particulado.

## INTRODUCTION

Masks are considered Personal Protective Equipment (PPE), and their function is to protect the respiratory system against chemical and biological occupational risks^(1)^.

In relation to chemical risks, these include particulate matter (PM), which contain different physical characteristics (size and number of particles, total surface area and electrostatic properties)^(2)^.

These chemical agents include metals, silica, Polycyclic Aromatic Hydrocarbons^(2)^, mineral matter (oxides of aluminum, calcium, silicon, titanium, iron, magnesium, manganese, sodium, and potassium), vapors, ethylene oxide, formaldehyde, glutaraldehyde, methyl methacrylate, gaseous by-products^(3)^, and secondary inorganic aerosols (sulfate, nitrate, and ammonium)^(4)^.

Biological hazards include bioaerosols (a mixture of viable and nonviable microorganisms), fungal spores, fragmented pollen^(5)^, bacteria, viruses, prions, and fungi. Therefore, the main purpose of wearing masks is to prevent the inhalation of airborne particles (natural or artificial) and biological organisms, as well as to trap these particles in the mask’s filters^(6)^.

Both chemical and biological exposure negatively affect the health and quality of life of those exposed, who may experience some damage such as acute or chronic poisoning, and prolonged exposure may cause damage to the nervous, hematopoietic or reproductive systems and even neoplastic pathologies^(7)^. Therefore, it is necessary that face masks have the appropriate filters for each type of exposure^(8)^.

Mask filter specifications include the inherent properties of the materials used in the mask, such as chemical composition of the filter and characteristics such as the thickness, density, and size of the fibers in the filter^(9)^.

Knowledge of the chemical and biological hazards present in healthcare setting and understanding the functions and specifications of mask filters are essential for healthcare professionals to effectively protect themselves and ensure a safe working setting for themselves and their patients. The correct selection and use of appropriate masks are cornerstones of occupational health and the prevention of work-related illnesses in healthcare.

Therefore, it is necessary to check whether the filters used are efficient in adsorbing the chemical component.

## OBJECTIVES

To assess the effectiveness of filters in filtering PM.

## METHODS

A systematic review (SR) of the literature was carried out, developed according to the recommendations of the JBI Manual for Evidence Synthesis^(10)^ for efficacy SR and Preferred Reporting Items for Systematic Review and Meta-Analyses (PRISMA) guidelines, a reference of the EQUATOR network^(10)^.

To begin this SR, it was necessary to understand the three stages to maintain methodological rigor: planning; execution; and reporting. Planning is the structural foundation for a well-executed study and consists of five stages: defining the team; choosing the topic; searching for previous SRs on the chosen topic; developing the research question; and grouping the information into the developed protocol^(11)^.

Therefore, the research question of this SR was developed using the PICOS acronym components for phenomenon. The question relates to any aspect of clinical practice, which generally involves a population (P) and an outcome (O), but not an intervention or comparator. Thus, P refers to population (filters), I to intervention, C to comparison, O to outcome (adsorption of chemical and biological particles), and S to study design (randomized clinical trial)^(12)^. The research question presented here is: which filters efficiently adsorb chemical and/or biological particles present in the air when tested in scientific interventions?

With the research question defined, a search was carried out on the International Prospective Register of Systematic Reviews platform, a free database of SR records maintained by the Centre of Reviews and Dissemination at the University of York and funded by the National Institute for Health Research^(13)^. Registration was carried out on this platform, under Protocol CRD42023445678^(14)^.

The protocol of this SR defined the following strategies: use of descriptors; use of their synonyms, in accordance with the Health Sciences Descriptors for the Latin American and Caribbean Literature in Health Sciences (LILACS) database; use of Medical Subject Headings (MeSH) descriptors in the Clarivate Analytics (Web of Science), Scopus, Medical Literature Analysis and Retrieval System Online (MEDLINE via PubMed), American Chemical Society, Royal Society Chemistry Sci Finder and Scientific Electronic Library Online (SciELO) databases; and combinations using controlled descriptors, keywords and Boolean operators AND and OR.

The search for studies was conducted in August and September 2023 in the MEDLINE databases via PubMed, Clarivate Analytics (Web of Science), Scopus, LILACS, American Chemical Society, Royal Society Chemistry, Sci Finder, and SciELO. The following MeSH terms were used, as shown in Chart 1: Filters, Adsorption, Organic Chemicals, Particulate Matter, Viruses, and Bacteria.

**Chart 1:** Search strategy in the databases included in the study, Brazil, 2023

Database	Search strategy
LILACS	((*filtros*) OR (*adsorção*)) AND (((“*material particulado*”) OR (“*compostos organicos*”) OR (virus) OR (bacteria))) AND ((“*ensaio clínico*”) OR (randomizado))
PubMed	((filters) OR (adsorption)) AND ((((“organic chemicals”) undefined (“particulate matter”)) undefined (viruses)) OR (bacteria)) Filters: “Clinical Trial”, “Randomized Controlled Trial”
SciELO	((filters) OR (adsorption) OR (adsorption)) AND ((“organic chemicals”) OR (“particulate matter”) OR (viruses) OR (bacteria)) AND (“clinical trial”) OR (“randomized controlled trial”)
Web of Science	(TS=(filters)) OR TS=(adsorption) AND (((TS=(“organic Chemicals”)) OR TS =(“particulate matter”)) OR TS= (viruses)) OR TS =(bacteria) AND (TS=(“clinical trial”)) OR TS=(“randomized controlled trial”)
Scopus	(filters) OR (adsorption) AND (“organic Chemicals”) OR (“particulate matter”) OR (viruses) OR (*bactéria*) AND (“clinical trial”)
American Chemical Society	(filters) OR (adsorption) AND (“organic Chemicals”) OR (“particulate matter”) OR (viruses) OR (*bactéria*) AND (“clinical trial”)
Royal Society Chemistry	(filters) OR (adsorption) AND (“organic Chemicals”) OR (“particulate matter”) OR (viruses) OR (*bactéria*) AND (“clinical trial”)
Sci Finder	(filters) OR (adsorption) AND (“organic Chemicals”) OR (“particulate matter”) OR (viruses) OR (*bactéria*) AND (“clinical trial”)

For this SR, original scientific intervention studies were included, whether randomized clinical trials or quasi-experimental studies, that investigated the effectiveness of respiratory protection devices or strategies in all types of settings. The selection process was not limited by time or language of publication, prioritizing the studies’ relevance and methodological rigor.

To extract data from the articles, we used the Ryyan reference manager, which created a spreadsheet to input data from the studies found. This tool excluded duplicate articles from the databases, while articles repeated in more than one database were retained in the database that returned the most articles in the search^(15)^.

Study selection was conducted by two independent reviewers, experts in the subject matter and members of the Study Group on Care Management, Scientific Publishing, and Workers’ Health (In Portuguese, *Grupo de Estudos em Gestão do Cuidado, Editoração Científica e Saúde do Trabalhador* - GeeST). Titles and abstracts were initially assessed, and after the reviewers’ agreement, the articles were read in full, with the inclusion and exclusion of selected studies.

Disagreements such as methodological quality, difficulty in determining the study’s objective and divergence in the study results were assessed and noted by a third reviewer with expertise in the subject studied and a participant in GeeST^(16)^.

To extract the data, a Microsoft Excel spreadsheet was used with the following data subtracted from the studies: filter; year; author; particles tested; interventions; main results; and article quality assessment.

Concerning the risk of bias, the Cochrane Collaboration Risk of Bias tool was used, which assesses the risk of bias through the synthesis of study results and no longer through the individual analysis of the study and its outcomes^(17)^.

The studies’ methodological quality included in this review was assessed using the JBI critical appraisal tool, and the level of evidence of included studies was classified as scientific evidence level 2, as all included studies must be interventional clinical trials, randomized or not (Gurevitch, 2018). The Cochrane Collaboration Risk of Bias 2.0 was used to assess the risk of bias^(17)^.

For meta-analysis, Jamovi’s Forest Plot tools and Higgins and Thompson’s I^2^ Statistic were used to determine the studies’ heterogeneity and the scientific evidence of this SR.

It was decided to present in the chart the results of included articles and only the articles that were assessed and included in the meta-analysis, as its objective is to present the best scientific evidence found on the subject.

Ethical review and approval were waived for this study, as no personal data or opinions of participants were used, as they assessed studies published in publicly available databases, and the study evaluators for the SR are experts who are part of the work team.

## RESULTS

**Figure 1:**
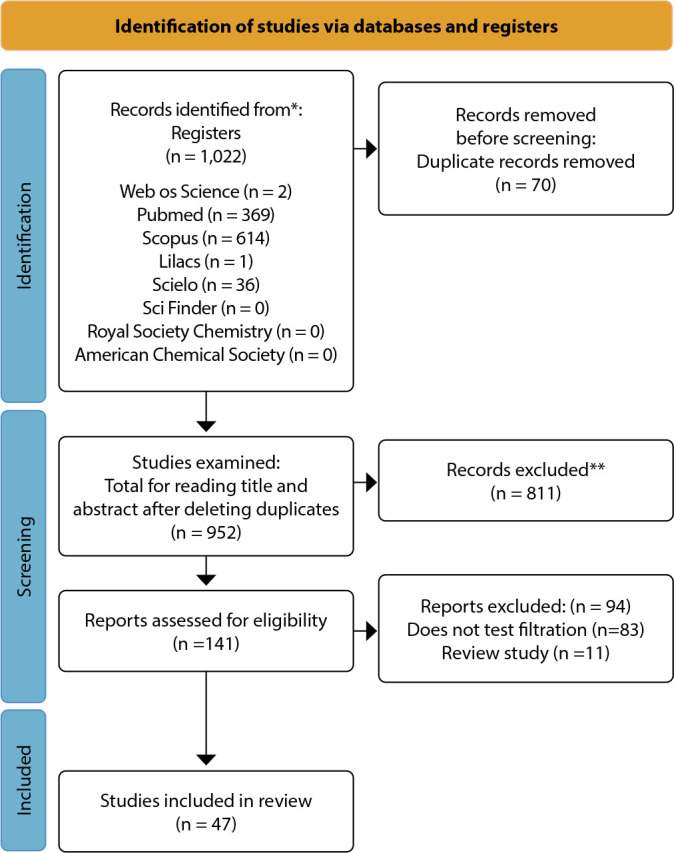
Flowchart according to the Preferred Reporting Items for Systematic Review and Meta-Analyses checklist, Brazil, 2020

*Source: The methodology for selecting articles for this review was informed and adapted from the Preferred Reporting Items for Systematic Reviews and Meta-Analyses flowchart. The figure above describes the flowchart for identifying, screening, and including the studies that form the basis of this analysis.*

Using the strategy and searching the selected databases, 1,022 articles from eight databases were included in this SR. Figure 1 demonstrates the identification, screening, eligibility, and inclusion of studies following PRISMA recommendations^(10)^.

Forty-seven studies were included in this SR. Chart 2 details the studies grouped for the meta-analysis, providing information on reference, filter, tested particles, interventions, main outcomes, and quality assessment (Brasil, 2023).

**Chart 2:** Characterization of meta-analysis studies (Brazil, 2023): reference, filter, particle size, interventions, main results, and quality

Title	Year Country	Authors	Main results	Design n=	Outcome	Article Quality Assessment (JBI)
A 01 A randomized double-blind crossover study of indoor air filtration and acute changes in cardiorespiratory health in a First Nations community	2012 Canada	Weichenthal S; Mallach G; Kulka R; Black A; Wheeler A; You H; St-Jean M; Kwiatkowski R; Sharp D.^(18)^	The results suggest that indoor PM2.5 may contribute to reduced lung function and that portable air filters may help alleviate these effects by effectively reducing indoor PM levels.	Randomized double-blind n=150	48 individuals participated in this study; EG: reduction in the median concentration of PM2.5 from 8 to 4 μg/m^3^; Reduction in particle number concentration from 7,669 to 5,352 particles/cm^3^, in addition to reduction in smoke and Polycyclic Aromatic Hydrocarbons.	11/13
A 02 Cardiopulmonary benefits of reducing indoor particles of outdoor origin: a randomized, double-blind crossover trial of air purifiers	2015 China	Chen R; Zhao A; Chen H; Zhao Z; Cai J; Wang C; Yang C; Li H; Xu X; Ha S; Li T; Kan H.^(19)^	This intervention study demonstrated clear cardiopulmonary benefits of indoor air purification among healthy young adults in a Chinese city with severe ambient particulate air pollution.	Double-blind randomized trial n= 35	35 students participated in the study; EG: reduction in PM2.5 concentration from 96.2 μg/m^3^ to 41.3 μg/m^3^.	12/13
A 03 Effect of Portable Air Filtration Systems on Personal Exposure to Fine Particulate Matter and Blood Pressure Among Residents in a Low-Income Senior Facility: A Randomized Clinical Trial	2018 USA	Morishita M; Adar SD; D’Souza J; Ziemba RA; Bard RL; Spino C; Brook RD.^(20)^	The results of this study showed that short-term use of portable air filtration systems reduced personal PM2.5 exposure and systolic BP among senior residents of a typical urban area in the United States. The use of these relatively inexpensive systems is potentially cardioprotective against PM2.5 exposure.	Randomized double-blind crossover trial n= 40	40 non-smoking adults; EG: reduction from 8.4 μg/m^3^ to 3.9 μg/m^3^ with the use of a low-efficiency HEPA filter; Reduction from 7.1 μg/m^3^ to 3.5 μg/m^3^ with the use of the high-efficiency HEPA filter.	12/13
A 04 Effects of filtered fresh air ventilation on classroom indoor air and biomarkers in saliva and nasal samples: A randomized crossover intervention study in preschool children	2019 China	Gao X; Xu Y; Cai Y; Shi J; Chen F; Lin Z; Chen T; Xia Y; Shi W; Zhao Z.^(21)^	The use of HEPA-HAVFs was effective in reducing PM2.5 in indoor classroom settings. Salivary lysozyme, as a nonspecific immunological biomarker, was significantly inversely associated with indoor PM2.5. Certain nasal bacteria may play important roles in mediating PM2.5 exposure and lysozyme levels in children.	Randomized crossover study n=43	43 children were analyzed in the study; EG: reduction of PM2.5 particles inside the classroom.	9/13
A 05 Particulate matter concentrations in residences: An intervention study evaluating stand-alone filters and air conditioners	2012 USA	Batterman S; Du L; Mentz G; Mukherjee B; Parker E; Godwin C; Chin JY; O’Toole A; Robins T; Rowe Z; Lewis T.^(22)^	The study shows that measurements over multiple seasons are necessary to characterize air quality and filter performance. The effectiveness of independent air filter interventions depends on occupant behavior, and strategies to ensure filter use should be an integral part of interventions.	Randomized clinical trial n=37	126 families with children with asthma; EG: there was no reduction in PM2.5.	13/13
A 06 Effectiveness of portable HEPA air cleaners on reducing indoor PM2.5 and NH3 in an agricultural cohort of children with asthma: A randomized intervention trial	2021 USA	Riederer, A.M.; Krenz, J.E.; TchongFrench, M.I.; Torres, E.; Perez, A.; Younglove, L.R.; Jansen, K.L.; Hardie, D.C.; Farquhar, S.A.; Sampson, P.D.; Karr, C.J.^(23)^	In this randomized trial of rural farm households, HEPA cleaners effectively reduced bedroom and living room PM2.5 levels by 60% and 42%, respectively, compared to asthma education alone, while significant ammonia reductions were not observed.	Randomized clinical trial n=71	69 families with children with poorly controlled asthma; EG: mean reduction of PM2.5 of 69% to 80%.	12/13
A 07 An indoor air filtration study in homes of elderly: cardiovascular and respiratory effects of exposure to particulate matter	2013 UE	Karottki DG; Spilak M; Frederiksen M; Gunnarsen L; Brauner EV.^(24)^	When comparing active and simulated indoor air filtration in the bedroom and living room for two weeks, we found no improvement in respiratory functional manovacuometry or lung function, nor detectable reduction in systemic inflammation, monocyte activation, or lung cell damage in this elderly population, including people using vasoactive medication but with relatively low baseline exposure levels.	Randomized double-blind crossover trial n=48	51 people were recruited; EG: 30% reduction in particle number concentration in the living room and close to 50% reduction in PM2.5 in the living room and bedroom.	12/13

*A - article; E - experimental; C - control; EG - experimental group; USA - United States of America; EU - European Union; PM - particulate matter; BP - blood pressure; HEPA - High Efficiency Particulate Arrestance.*

We chose to analyze the seven articles comprising the meta-analysis because we considered this to represent the most comprehensive and up-to-date synthesis of literature relevant to our research question. By focusing on the studies already compiled and assessed in this meta-analysis, we sought to leverage a consolidated evidence base to inform our analyses.

Most of the articles included in this SR met the methodological quality assessment criteria, according to the JBI for randomized clinical trials. Of the 47 articles analyzed, 44 (93.62%) were considered to be of good quality, presenting clear answers to methodological questions. However, three articles (6.38%) require additional information for a complete assessment, as they presented gaps such as: treatment group assignment was not clearly described; blinding of treatment groups was not evident; it was unclear whether outcome assessors were blinded to treatment assignment; participant follow-up was not complete; differences between groups in terms of follow-up were not adequately described and analyzed; and outcomes were not measured in the same way for treatment groups.

**Figure 2:**
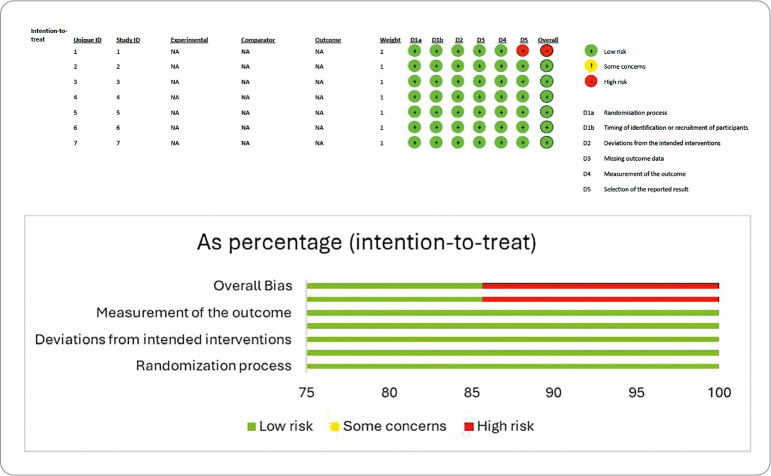
Assessment of the risk of bias of studies included in the systematic review according to the Cochrane Collaboration Risk of Bias tool, Brazil, 2023

*Source: Cochrane Collaboration Risk of Bias tool^(25)^.*

Despite these specific observations, the articles included in this SR were considered to be of good quality, with most studies demonstrating methodological rigor. Regarding the risk of bias assessed using the Cochrane Collaboration Risk of Bias tool, the results are presented in Figure 2.

To determine the risk of bias, we used this tool, which synthesizes study results, moving away from the isolated analysis of each study and its outcomes. Its structure consists of five domains, which contain “signal questions”-critical questions that provide additional data for assessment. The answer options for these questions are “yes”, “probably yes”, “probably not”, “no”, “no information”, and “not applicable”. It is worth noting that assertive answers such as “yes” and “no” often indicate the existence of robust evidence. The “not applicable” option, in turn, is only available for optional questions. As users interact with the tool, responses are processed by an algorithm that ultimately categorizes the risk of bias for each domain as high, low, or with some concern^(25)^, as shown in Figure 2.

This assessment demonstrated that seven studies accounted for 14.87% of them were defined as unclear; six (12.75%) studies had a high risk of bias; and 34 (72.38%) studies had a low risk of bias.

**Figure 3:**
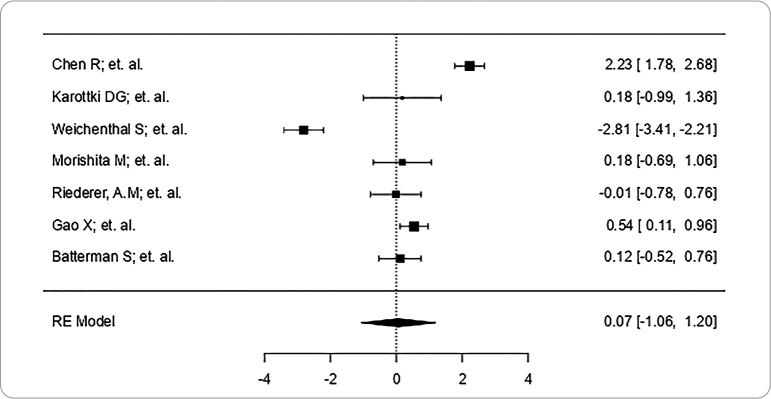
Forest plot graph presenting the meta-analysis of the homogeneous studies used in this study, Brazil, 2024

*Source: Jamovi’s Floret Plot tool.*

Figure 3 presents the meta-analysis of the articles selected using the Forest Plot tool.

The meta-analysis carried out in the study^(26)^ brings statistical significance to the study, as it showed a high scientific evidence index, in which the lower limit is 2.23, with a Confidence Interval between 1.78 and 2.68 and an upper limit of 2.68, showing that the scientific evidence is weak, as the studies are similar in their measurements, but the meta-analysis showed heterogeneity of the data.

Despite the studies’ homogeneity, the meta-analysis revealed significant heterogeneity between them, indicating that the variability is not random.

## DISCUSSION

In studies included in this SR, it was demonstrated that filtration using the High Efficiency Particulate Arrestance (HEPA) filter reduces PM concentrations by 70% to 80% (in particles of varying sizes) in field applications^(17)^.

Statistical significance is observed in the study, as it showed a high scientific evidence index according to the Forest Plot graph. A modest but statistically significant decrease in blood pressure (BP) was found after the intervention. This finding contrasts with that of several previous studies on air filtration in countries with cleaner air, suggesting that these cardiovascular benefits may be more easily achieved in regions with severe air pollution problems^(26)^.

The effectiveness of air filters depends on the type of pollutant to be filtered; therefore, an alternative choice is the HEPA filter, which captures 99.97% of the particles present in the air, or the Ultra Particulate Air (ULPA) filter, which captures 99.99% of these particles, representing a safe alternative for protection against chemical and biological risks^(8)^.

In the mechanism of adsorption of chemical and biological particles on mask filters, particles are attracted to the filter surface by electrostatic forces, Van der Waals forces or hydrophobic forces^(27)^. Electrostatic forces are stronger and responsible for the adsorption of positively or negatively charged particles^(27)^. Masks are generally made of materials that have a negative electrical charge, which helps attract positively charged particles such as dust, pollen, and viruses. Each mask has specific characteristics and purposes, many of which utilize electrostatic properties to increase filtration efficiency. The most common masks used by healthcare professionals are surgical masks, which filter the air exhaled by the user to protect the setting and, to a lesser extent, filter the inhaled air from larger droplets^(27)^.

N95 or PFF2 respirators, which are disposable half-mask respirators that fit snugly against the face, filter at least 95% of airborne particles, including those as small as 0.3µm. They have multiple layers, many of which are electrostatically treated to attract particles. PFF3, the highest class of disposable particle filters, with a minimum efficiency of 99.95% of 0.6 µm particles, protects workers in settings with highly toxic substances, handling radioactive materials and areas of high biological risk^(28)^.

Hydrophobic forces are forces that repel water, and masks are often made of materials that are hydrophobic, which helps attract hydrophilic particles such as water vapor and droplets. Particle size also affects their adsorption on mask filters, with larger particles being more easily attracted to the filter surface than smaller particles. This is because larger particles have more surface area to interact with adsorption forces^(29)^.

The efficiency of a mask filter in adsorbing chemical and biological particles depends on several factors, including the type of filter material, the filter surface properties, the particle size, and the particle concentration^(30)^.

The masks are made using the melt blown technique, a process in which electrical charges are injected into the material, creating a nearly permanent electrical field that provides sufficient PM filtration through an electrostatic force. Non-woven fibrous substances, such as polypropylene, polyethylene, polylactic acid and polytetrafluoroethylene (PTFE), are the most studied materials^(30)^.

Melt-blown polypropylene nonwoven fibers have stable properties, fine fiber diameter (0.5-4 μm), high porosity, good air permeability, and good filtration resistance. Therefore, the performance of filter materials prepared with polypropylene is much better than that of other materials^(27)^.

A randomized, double-blind, three-way crossover intervention study, with filtration using the low-efficiency (LE) HEPA and high-efficiency (HE) HEPA filters of ambient air using air purification systems in the bedroom and main living area of residences, showed that the LE filter removes 99% of particles with a size of 2.0 μm, while the HE filter removes 99.97% of particles with a size of 0.3 μm, complementing that this practical and relatively inexpensive approach can be an effective tool to reduce health effects related to PM2.5^(20)^.

The HEPA filter is an air filtration system widely used in settings where air quality is critical. It is capable of retaining a wide variety of particles, including small ones, making it essential in many applications. They operate through three main mechanisms to retain particles: interception: particles that pass too close to a filter fiber are physically trapped and adhere to the fiber; inertial impact: larger particles cannot avoid the filter fibers and collide with them due to their mass, becoming trapped; diffusion (Brownian motion): ultrafine particles (smaller than 0.1 µm) perform an erratic random movement (Brownian motion) due to collisions with gas molecules. This movement increases the likelihood of collision with the filter fibers and capture^(31)^.

The use of HEPA filters in hospital settings is crucial to reduce the incidence of hospital-acquired infections caused by airborne bacteria, viruses, and fungi. They remove pathogens that cause diseases such as tuberculosis, influenza, and other respiratory infections, contributing to the safety of patients and healthcare professionals. Therefore, the use of HEPA filters contributes to better indoor air quality by eliminating fine particles that can be inhaled and cause long-term health problems^(32)^.

Evidence also shows that wearing N-95 face masks for two hours during peak traffic results in reductions in exhaled nitric oxide, multiple inflammatory cytokines in exhaled breath condensate, but no clear beneficial effect on endothelial or systemic function, with low evidence that wearing this type of PPE reduces systemic inflammation or oxidative stress, evidencing here that they are sealed and well-fitting masks^(33)^.

The N-95 classification is designated by a minimum filtration efficiency of 95%, composed of several layers of non-woven polypropylene fabric. The efficiency of this model is determined by the filtration capacity, caused by the electrostatic charge provided by the polypropylene fiber, whose action is essential to guarantee the protection of the wearer^(28)^.

A study examined the impact of indoor air filtration on acute changes in clinical measures of respiratory and cardiovascular health in a First Nations community. This first crossover study was conducted on a First Nations reserve in Manitoba, Canada, and included 37 residents in 20 homes. A study examining air quality in First Nations communities in Manitoba, Canada, using a 3M Filtrete FAP03-RS Ultra Clean Air Purifier electrostatic air filter and a placebo filter, found improvements in measures of lung function, BP, and endothelial function^(18)^.

Another study, using respirators with a 3M Filtrete particle filter model FAP04, may produce cardiovascular benefits by improving autonomic nervous function and reducing BP. The study was conducted at the Fudan University, Fenglin campus, located in the central urban area of Shanghai. This intervention was designed as a randomized, double-blind, crossover trial^(34)^.

The ten rooms were randomized into two groups of five rooms each. One group used an air purifier placed in the center of the room for 48 hours, corresponding to two weekends, followed by a two-week washout period, and then used a dummy air purifier under the same conditions for another 48 hours. The other group simply reversed the order in which the real and dummy air purifiers were used. All rooms used the same qualified air purifiers (model FAP04, 3M Filtrete, Shanghai, China), with the only difference being the removal of the filter screen on the dummy purifiers. The air purifiers’ automatic air pollution detection feature was disabled in both groups. All participants and the research team were blinded to group assignment^(34)^.

A prospective, randomized, unblinded crossover study of 22 healthy Asian volunteers (nine men and 13 women) compared the proportion of particle counts inside and outside two oxygen masks: the FiltaMask and a Hudson-type mask (Well Lead Medical, Guangzhou, China). The results demonstrated that the FiltaMask reduced the concentration of particles from 0.02 μm to 12 μm inside the mask relative to ambient concentration. The degree of reduction was significantly greater than that observed with a Hudson-type mask, indicating that the reduction was not simply due to particle concentration dilution by oxygen^(35)^.

Forty-three children aged 5 to 14 years from Songjiang, China, a suburb of Shanghai, were recruited to participate in a double-blind, randomized, crossover study. Time-integrated PM2.5 samples collected over 48 hours were collected on 37 mm PTFE (Teflon) filters using personal environmental monitors (PEMs; SKC, USA) with AirChek XR5000 pumps (SKC, USA). The indoor air filter was found to reduce the measured oxidative potential of indoor and personal PM exposure. Using source contributions from the EPA MPF 5.0 model, the sources of PM2.5 elements that infiltrated into the indoor environment and were reduced indoors by an air filter were identified. The indoor air filter was found to reduce the measured oxidative potential of personal PM exposure^(36)^.

Although the aerosol properties of bioaerosols are generally considered similar to those of non-bioaerosols, respirator selection and maintenance are complicated by their biological nature. Bioaerosols include bacteria, viruses, fungi, algae, and dust mites. Additionally, biological products such as pollen, endotoxins, proteins, and animal excrement form aerosols. Both viable and non-viable forms of bioaerosols can be hazardous to health. Infectious bioaerosols produce adverse health effects due to their ability to incubate, grow, multiply, and produce toxic substances^(37)^.

### Study limitations

The study provides preliminary evidence on the effectiveness of HEPA filters in removing PM. However, further research is needed to confirm and expand on these results, given the complexity of the topic and the diversity of factors that can influence air quality.

### Contributions to nursing

This research assessed the effectiveness of different filters in removing PM of various sizes in simulated and non-simulated healthcare settings. Additionally, the results will be used to develop a new HeLP mask filter, an ergonomic and sustainable mask made from disinfectable material, which will undergo rigorous testing for protection against chemical and biological risks relevant to healthcare workers. Essential data will be provided for selecting ideal filters, optimizing maintenance protocols, and, crucially, informing the development of more effective personal protective technologies for healthcare workers.

This mask will protect healthcare workers against certain diseases, such as allergic rhinitis, hypothyroidism, migraines, asthma, high BP, systemic hypertension, fibromyalgia, and even malignant neoplasms.

In this regard, NR32, which addresses protection against occupational chemical hazards to which healthcare workers are exposed, becomes relevant. The creation and implementation of appropriate institutional policies and regulations are essential to protect healthcare workers.

## CONCLUSIONS

The results of this study indicate that HEPA filtration was superior in reducing PM in ambient air (99.97% capture). Although the ULPA filter has a slightly higher capture (99.99%), both represent safe alternatives against chemical and biological risks.

Other filters besides HEPA, such as 37 mm PTFE (Teflon) filters, were also tested, showing a reduction in the oxidative potential of internal exposure to PM2.5. The FiltaMask N-95 and Hudson filters reduced particles from 0.02 µm to 1 µm inside the mask. And the 3M Filtrete FAP03-RS Ultra Clean Air Purifier masks reduced internal PM levels, alleviating the effects of pollution.

## Data Availability

The research data are available within the article.

## References

[1] Universidade Federal do Espírito Santo (2004). Máscaras de Proteção Respiratória: N95, PFF1, PFF2 ou PFF3?.

[2] Filho PA. (2023). Asma brônquica: asma e poluição atmosférica.

[3] Mohanty A, Kabi A, Mohanty AP. (2019). Health problems in healthcare professionals: a review. J Family Med Prim Care.

[4] Morakinyo OM, Mokgobu NI, Mukhlifa MS, Hunter RP. (2016). Health outcomes of exposure to biological and chemical components of inhalable and respirable particles. Int J Environ Res Public Health.

[5] Zhai Y, Li X, Wang T, Wang B, Li C, Zeng G. (2018). A review on airborne microorganisms in particulate matters: composition, characteristics and influence factors. Environ Int.

[6] McDonald F, Claire J. Horwell, Wecker Richard, Dominelli Lena, Loh Miranda (2020). Facemask use for community protection from air pollution disasters: an ethical overview and framework to guide agency decision making. Int J Disaster Risk Reduct.

[7] Charlier B, Coglianese A, Rosa F, Caro F, Piazza O, Motta O (2021). Chemical risk in hospital settings: overview on monitoring strategies and international regulatory aspects. J Public Health Res.

[8] Kadam VV, Wang L, Padhye R. (2016). Electrospun nanofibre materials to filter air pollutants: a review. J Ind Text.

[9] Conselho Nacional de Saúde (CNS) (2020). Recomendação nº 072, de 21 de dezembro de 2020. Recomenda a distribuição obrigatória a todas as pessoas, pela rede SUS, de máscaras adequadas e reutilizáveis, para fazer frente às necessidades emergenciais da população diante da pandemia da COVID-19.

[10] Aromataris E, Lockwood C, Porritt K, Pilla B, Jordan Z (2024). JBI Manual for Evidence Synthesis. JBI;.

[11] Page MJ, McKenzie JE, Bossuyt PM, Boutron I, Hoffmann TC, Mulrow CD (2021). The PRISMA 2020 statement: an updated guideline for reporting systematic reviews. BMJ.

[12] Canto GL. (2020). Revisões sistemáticas da literatura: guia prático.

[13] Ministério da Saúde (BR) (2021). Diretrizes Metodológicas: elaboração de revisão sistemática e meta-análise de ensaios clínicos e randomizados.

[14] National Institute for Health and Care Research (NIHR) (2024). PROSPERO (International Prospective Register of Systematic Reviews).

[15] Teixeira EP, Lynn FA, Souza ML. (2024). A Guide for Systematic reviews of observational studies. Texto Contexto Enferm.

[16] Rodrigues CL, Ziegelmann PK. (2011). Metanálise: um guia prático. Clin Biomed Res.

[17] Du L, Batterman S, Parker E, Godwin C, Chin JY, O’Toole A (2011). Particulate matter concentrations and filter effectiveness of air purifiers in permanent supportive housing in Detroit, Michigan. Constr Build Environ.

[18] Weichenthal S, Mallach G, Kulka R, Black A, Wheeler A, You H (2012). A randomized double-blind crossover study of indoor air filtration and acute changes in cardiorespiratory health in a First Nations community. Environ Health Perspect.

[19] Chen R, Zhao A, Chen H, Zhao Z, Cai J, Wang C (2015). Cardiopulmonary benefits of reducing indoor particles of outdoor origin: a randomized, double-blind, crossover trial of air purifiers. J Am Coll Cardiol.

[20] Morishita M, White LF, Minter T, Sacks J, Zamore M, Cohen SA (2018). Effect of portable air filtration systems on personal fine particulate matter exposure and blood pressure among residents in a low-income elderly facility: a randomized crossover intervention trial. J Am Geriatr Soc.

[21] Gao X, Xu Y, Cai Y, Shi J, Chen F, Lin Z (2019). Effects of filtered fresh air ventilation on classroom indoor air and biomarkers in nasal and saliva samples: a randomized crossover intervention study in preschool children. Environ Res.

[22] Batterman S, Du L, Mentz G, Mukherjee B, Parker E, Godwin C (2012). Particulate matter concentrations in homes: a randomized study evaluating stand-alone filters and air conditioners. Indoor Air.

[23] Riederer AM, Krenz JE, Tchong-French MI, Torres E, Perez A, Younglove LR (2021). Efficacy of portable HEPA air cleaners in reducing indoor PM2.5 and NH3 in a farmworker cohort of children with asthma: a randomized intervention trial. Indoor Air.

[24] Karottki DG, Spilak M, Frederiksen M, Gunnarsen L, Brauner EV, Kolarić B (2013). An indoor air filtration study in homes of elderly subjects: cardiovascular and respiratory effects of particulate matter exposure. Environ Health.

[25] Cochrane Methods (2019). RoB 2: Revised Cochrane risk-of-bias tool for randomized trials.

[26] Miao H, Liu J, Li P, Hu Q, Huang W, Zheng X (2021). Effects of air filtration interventions on cardiovascular health: a systematic review and meta-analysis of randomized controlled trials. Environ Sci Technol.

[27] Wang AB, Zhang X, Gao LJ, Zhang T, Xu HJ, Bi YJ. (2023). A review of filtration performance of protective masks. Int J Environ Res Public Health.

[28] Secretaria da Saúde do Ceará (SESA) (2022). Nota Informativa sobre Máscaras.

[29] Zhang JC. (2021). Interfacial assembly of two-dimensional MXenes. J Energy Chem.

[30] Associação Brasileira de Normas Técnicas (ABNT) (2011). NBR 13698 Equipamento de proteção respiratória - Peça semifacial filtrante para partículas - Requisitos, ensaios e marcação.

[31] Science Direct Engineerig (2024). High-Efficiency Particulate Air Filter.

[32] Santos RA, Silva MAS, Marciano FR, Pereira MR, Hasslocher-Moreno AM. (2025). Microbiological Efficiency of HEPA Filtration Systems in Preventing Multidrug-Resistant Bioaerosols in Hospital Environments. Rev Gestão Secret.

[33] Jiang M, Wang Q, Huang B, Wang Y, Hu F, Hu M (2021). The effects of face mask usage on the cardiopulmonary system of healthy young adults: a double-blind, randomized, crossover study. Int J Hyg Environ Health.

[34] Cui X, Li F, Xiang J, Fang L, Chung MK, Day DB (2018). Cardiopulmonary effects of overnight indoor air filtration in healthy non-smoking adults: a double-blind randomized crossover study. Environ Int.

[35] Wai JKM, Gomersall CD. (2011). A controlled crossover study in human volunteers on the in vivo filtration efficacy of a high-efficiency particulate air filter oxygen mask system. Am J Infect Control.

[36] Brehmer C, Norris C, Barkjohn K, Feng Y, Sacks J, Zamore M (2020). The impact of residential air cleaners on PM2.5 oxidative potential and the role of associated metals and sources from indoor and outdoor exposure. Environ Res.

[37] Kim KH, Kabir E, Jahan SA. (2018). Airborne bioaerosols and their impact on human health. J Environ Sci (China).

