# Supplementary material for: Effectiveness of particulate matter filtration: systematic review and meta-analysis
Source: Rev Bras Enferm. 2026 Mar 16;79:e20240454. doi: 10.1590/0034-7167-2024-0454 (PMC12991437; doi:10.1590/0034-7167-2024-0454)
Supplement: Translation

# Translation: Efetividade da filtração de material particulado: revisão sistemática e metanálise

**DOI:** 10.1590/0034-7167-2024-0454pt

**Authors:** Francieli Faustino, Helenize Ferreira Lima Leachi, Aline Franco da Rocha, Ana Paula Silva, Rosângela Marion da Silva, Renata Perfeito Ribeiro

**Affiliations:** I Universidade Estadual de Londrina, Londrina, Paraná, Brasil; II Universidade Federal de Santa Maria. Santa Maria, Rio Grande do Sul, Brasil

**Keywords:** Filtros, Adsorção, Compostos Químicos, Efetividade, Compostos Orgânicos

## Abstract

**Objetivos:**

avaliar a efetividade dos filtros na filtração de material particulado.

**Métodos:**

revisão sistemática da literatura com a pergunta de pesquisa: quais filtros adsorvem partículas químicas e/ou biológicas presentes no ar de forma eficiente quando testados em intervenções científicas? As bases de dados utilizadas foram *Medical Literature Analysis and Retrieval System Online, Web of Science*, Scopus, Literatura Latino-Americana e do Caribe em Ciências da Saúde, *American Chemical Society, Royal Society Chemistry, Sci Finder* e *Scientific Eletronic Library Online*.

**Resultados:**

foram incluídos nesta revisão 47 estudos. O filtro *High Efficiency Particulate Arrestance* foi testado em 36 estudos. Os sete estudos utilizados para realização da metanálise foram homogêneos.

**Conclusões:**

a filtração realizada com o uso do filtro *High Efficiency Particulate Arrestance* reduziu concentrações de material particulado em 70% a 80% nas avaliações e intervenções apresentadas, contribuindo para evidências que esse filtro reduz as concentrações de material particulado.

## INTRODUÇÃO

As máscaras são consideradas Equipamentos de Proteção Individual (EPI), e têm a função de proteger o sistema respiratório contra riscos ocupacionais químicos e biológicos^(1)^.

Em relação aos riscos químicos, incluem-se os materiais particulados (MPs), que contêm diferentes características físicas (tamanho e número de partículas, área superficial total e propriedades eletrostáticas)^(2)^.

Entre esses agentes químicos podem-se citar metais, sílica, Hidrocarbonetos Policíclicos Aromáticos^(2)^, matéria mineral (óxidos de alumínio, cálcio, silício, titânio, ferro, magnésio, manganês, sódio e potássio), vapores, óxido de etileno, formaldeído, glutaraldeído, metacrilato de metila, subprodutos gasosos^(3)^ e aerossóis inorgânicos secundários (sulfato, nitrato e amônio)^(4)^.

Sobre os riscos biológicos, podem-se citar bioaerossóis (mistura de microrganismos viáveis e inviáveis), esporos de fungos, pólen fragmentado^(5)^, bactérias, vírus, príons e fungos. Portanto, o principal objetivo do uso de máscaras é prevenir a inalação de partículas transportadas pelo ar (naturais ou artificiais) e organismos biológicos, além de reter essas partículas nos filtros das máscaras^(6)^.

Tanto a exposição química quanto a biológica afetam negativamente a saúde e a qualidade de vida dos expostos, que poderão apresentar alguns danos como intoxicação aguda ou crônica, e a exposição prolongada pode acarretar danos no sistema nervoso, hematopoiético ou reprodutivo e ainda patologias neoplásicas^(7)^. Portanto, é necessário que as máscaras faciais possuam os filtros adequados para cada tipo de exposição^(8)^.

As especificações do filtro das máscaras incluem as propriedades inerentes dos materiais usados na máscara, como a composição química do filtro e características como espessura, densidade e tamanho das fibras no filtro^(9)^.

O conhecimento sobre os riscos químicos e biológicos presentes no ambiente de saúde e a compreensão das funções e das especificações dos filtros das máscaras são indispensáveis para que os trabalhadores da saúde possam se proteger de forma eficaz e garantir um ambiente de trabalho seguro para si e para os pacientes. A escolha e o uso correto das máscaras adequadas são pilares da saúde ocupacional e da prevenção de doenças relacionadas ao trabalho na área da saúde.

Dessa maneira, faz-se necessário verificar se os filtros utilizados são eficientes na adsorção do componente químico.

## OBJETIVOS

Avaliar a efetividade dos filtros na filtração de material particulado.

## MÉTODOS

Foi realizada revisão sistemática (RS) da literatura, desenvolvida de acordo com as recomendações do JBI *Manual for Evidence Synthesis* ^(10)^ para RS de eficácia e das diretrizes do *checklist Preferred Reporting Items for Systematic Review and Meta-Analyses* (PRISMA) referencial da rede EQUATOR^(10)^.

Para iniciar esta RS, foi necessária a compreensão das três etapas a manter o rigor metodológico: planejamento; execução; e relato. O planejamento é a base estrutural para que o estudo seja bem executado, e é composto por cinco etapas: definição da equipe; escolha do tópico; busca por RS anteriores no tópico escolhido; elaboração da pergunta de pesquisa; e agrupamento das informações no protocolo elaborado^(11)^.

Portanto, a pergunta de pesquisa desta RS foi elaborada utilizando os componentes do acrônimo PICOS de fenômeno, a questão se relaciona a qualquer aspecto da prática clínica, que geralmente envolve uma população (P) e um desfecho (O), mas não uma intervenção ou comparador. Assim, P refere-se à população (filtros), I, à intervenção, C, à comparação, O, ao desfecho (adsorção de partículas químicas e biológicas), e S, ao tipo de estudo (ensaio clinico e randomizado)^(12)^, apresentando como pergunta de pesquisa: quais filtros adsorvem partículas químicas e/ou biológicas presentes no ar de forma eficiente quando testados em intervenções científicas?

Com a pergunta de pesquisa definida, realizou-se uma busca na plataforma *International Prospective Register of Systematic Reviews*, uma base de registros de RS, gratuita, mantida pelo *Centre of Reviews and Dissemination* da Universidade de York e financiada pelo *National Institute for Health Research* ^(13)^. Foi realizado o cadastrado nessa plataforma, sob Protocolo n^o^ CRD42023445678^(14)^.

O protocolo desta RS definiu as seguintes estratégias: uso de descritores; uso de seus sinônimos, em concordância com os Descritores em Ciências da Saúde para a base de dados Literatura Latino-Americana e do Caribe em Ciências da Saúde (LILACS); uso de descritores da *Medical Subject Headings* (MeSH) nas bases de dados *Clarivate Analytics* (*Web of Science*), Scopus, *Medical Literature Analysis and Retrieval System Online* (MEDLINE via PubMed), *American Chemical Society, Royal Society Chemistry Sci Finder* e *Scientific Eletronic Library Online* (SciELO); e combinações empregando descritores controlados, palavras-chave e operadores booleanos *AND* e *OR*.

A busca pelos estudos foi realizada em agosto e setembro de 2023 nas bases de dados MEDLINE via PubMed, *Clarivate Analytics* (*Web of Science*), Scopus, LILACS, *American Chemical Society, Royal Society Chemistry, Sci Finder* e SciELO. Utilizaram-se os seguintes termos MeSH, conforme apresentado no Quadro 1: *Filters, Adsorption, Organic Chemicals, Particulate Matter, Viruses* e *Bacteria*.

**Quadro 1:** Estratégia de busca nas bases de dados inseridas no estudo, Brasil, 2023

Base de dados	Estratégia de busca
LILACS	((filtros) *OR* (adsorção)) *AND* (((“material particulado”) *OR* (“compostos organicos”) *OR* (*virus*) *OR* (*bacteria*))) *AND* ((“ensaio clínico”) *OR* (randomizado))
PubMed	((*filters*) *OR* (*adsorption*)) *AND* ((((“*organic chemicals*”) *undefined* (“*particulate matter*”)) *undefined* (*viruses*)) *OR* (*bacteria*)) *Filters:* “*Clinical Trial*”, “*Randomized Controlled Trial*”
SciELO	((*filters*) *OR* (*adsorption*) *OR* (*adsorption*)) *AND* ((“*organic chemicals*”) *OR* (“*particulate matter*”) *OR* (*viruses*) *OR* (*bacteria*)) *AND* (“*clinical trial*”) *OR* (“*randomized controlled trial*”)
*Web of Science*	(TS=(*filters*)) *OR* TS=(adsorption) *AND* (((TS=(“*organic Chemicals*”)) *OR* TS =(“*particulate matter*”)) *OR* TS= (*viruses*)) *OR* TS =(*bacteria*) *AND* (TS=(“*clinical trial*”)) *OR* TS=(“*randomized controlled trial*”)
Scopus	(*filters*) *OR* (*adsorption*) *AND* (“*organic Chemicals*”) *OR* (“*particulate matter*”) *OR* (*viruses*) *OR* (bactéria) *AND* (“*clinical trial*”)
*American Chemical Society*	(*filters*) *OR* (*adsorption*) *AND* (“*organic Chemicals*”) *OR* (“*particulate matter*”) *OR* (*viruses*) *OR* (bactéria) *AND* (“*clinical trial*”)
*Royal Society Chemistry*	(*filters*) *OR* (*adsorption*) *AND* (“*organic Chemicals*”) *OR* (“particulate *matter*”) *OR* (*viruses*) *OR* (bactéria) *AND* (“*clinical trial*”)
*Sci Finder*	(*filters*) *OR* (*adsorption*) *AND* (“*organic Chemicals*”) *OR* (“*particulate matter*”) *OR* (*viruses*) *OR* (bactéria) *AND* (“*clinical trial*”)

Para esta RS, foram incluídos estudos científicos originais de intervenção, sejam eles ensaios clínicos randomizados ou estudos quase-experimentais, que investigassem a eficácia de dispositivos ou estratégias de proteção respiratória em todos os tipos de ambientes. A seleção não apresentou limites de tempo ou idioma de publicação, priorizando a relevância e o rigor metodológico dos estudos.

Para extração dos dados dos artigos, foi utilizado o gerenciador de referências Ryyan, no qual foi elaborada uma planilha para inserir os dados dos estudos encontrados. Nesta ferramenta, foi realizada a exclusão dos artigos duplicados nas bases de dados, onde os artigos repetidos em mais de uma base de dados foram mantidos na base de dados que apresentou mais artigos na busca realizada^(15)^.

A seleção dos estudos foi realizada por dois revisores independentes especialistas no assunto em estudo e participantes do Grupo de Estudos em Gestão do Cuidado, Editoração Científica e Saúde do Trabalhador (GeeST). Inicialmente, foi realizada a avaliação dos títulos e resumos, e após a concordância dos avaliadores, foi realizada a leitura dos artigos na íntegra, com inclusão e exclusão dos estudos encontrados.

Discordâncias como qualidade metodológica, dificuldade de determinar o objetivo do estudo e divergência nos resultados do estudo foram avaliadas e anotadas por um terceiro revisor com *expertise* no assunto estudado e participante do GeeST^(16)^.

Para extração dos dados, foi utilizada uma planilha do *Microsoft Excel* com os seguintes dados subtraídos dos estudos: filtro; ano; autor; partículas testadas; intervenções; principais resultados; e avaliação da qualidade dos artigos.

Em relação ao risco de viés, foi utilizada a ferramenta *Cochrane Collaboration Risk of Bias*, que avalia o risco de viés por meio da síntese dos resultados do estudo e não mais pela análise individual do estudo e seus desfechos^(17)^.

A qualidade metodológica dos artigos incluídos nesta revisão foi avaliada com o instrumento de avaliação crítica do JBI e o nível de evidência dos estudos incluídos. Foi classificada como 2 de evidência científica, pois todos os estudos incluídos precisam ser ensaios clínicos com intervenção, aleatorizados ou não (Gurevitch, 2018). Para a avaliação do risco de viés, foi utilizada a *Cochrane Collaboration Risk of Bias* 2.0^(17)^.

Para metanálise, foram utilizadas as ferramentas *Forest Plot* do Jamovi e a Estatística I^2^ de Higgins e Thompson para determinar a heterogeneidade dos estudos e a evidência científica desta RS.

Optou-se por apresentar no quadro resultados dos artigos incluídos e somente os artigos que foram avaliados e incluídos na metanálise, pois seu objetivo é apresentar as melhores evidências científicas encontradas sobre o assunto.

A revisão ética e aprovação foram dispensadas para este estudo, pois não foram utilizados dados pessoais ou opiniões de participantes, pois avaliam estudos publicados em bases de dados de conhecimento público, e os avaliadores dos estudos para a RS são *experts* que fazem parte da equipe de trabalho.

## RESULTADOS

Com a estratégia e busca nas bases de dados selecionadas, foram incluídos nesta RS 1.022 artigos em oito bases de dados. Na Figura 1, demonstram-se a identificação, triagem, elegibilidade e inclusão dos estudos seguindo as recomendações do PRISMA^(10)^.

**Figura 1:** Fluxograma segundo o *checklist Preferred Reporting Items for Systematic Review and Meta-Analyses,* Brasil, 2020

*Fonte: a metodologia de seleção dos artigos para esta revisão foi informada e adaptada do fluxograma Preferred Reporting Items for Systematic Reviews and Meta-Analyses. A figura acima descreve o fluxo de identificação, triagem e inclusão dos estudos que compõem a base desta análise.*

Quarenta e sete estudos foram incluídos nesta RS. O Quadro 2 detalha os estudos agrupados para a metanálise, fornecendo informações sobre referência, filtro, partículas testadas, intervenções, principais resultados e avaliação da qualidade (Brasil, 2023).

**Quadro 2:** Caracterização dos estudos da metanálise (Brasil, 2023): referência, filtro, tamanho das partículas, intervenções, principais resultados e qualidade

Título	Ano País	Autores	Principais resultados	Delineamento n=	Desfecho	Avaliação da qualidade dos artigos (JBI)
A 01 *A randomized double-blind crossover study of indoor air filtration and acute changes in cardiorespiratory health in a First Nations community*	2012 Canadá	Weichenthal S; Mallach G; Kulka R; Black A; Wheeler A; You H; St-Jean M; Kwiatkowski R; Sharp D.^(18)^	Os resultados sugerem que o MP2,5 em ambientes fechados pode contribuir para redução da função pulmonar e que filtros de ar portáteis podem ajudar a aliviar esses efeitos, reduzindo efetivamente os níveis internos de material particulado.	Randomizado duplo-cego n=150	48 indivíduos participaram desse estudo; GE: redução da concentração mediana de MP2,5 de 8 para 4 *μg*/m^3^; Redução da concentração do número de partículas de 7.669 para 5.352 partículas/cm^3^, além de redução de fumaça e Hidrocarbonetos Policíclicos Aromáticos.	11/13
A 02 *Cardiopulmonary benefits of reducing indoor particles of outdoor origin: a randomized, double-blind crossover trial of air purifiers*	2015 China	Chen R; Zhao A; Chen H; Zhao Z; Cai J; Wang C; Yang C; Li H; Xu X; Ha S; Li T; Kan H.^(19)^	Este estudo de intervenção demonstrou benefícios cardiopulmonares claros da purificação do ar interno entre adultos jovens e saudáveis ​​em uma cidade chinesa com poluição atmosférica ambiental grave por partículas.	Ensaio randomizado duplo-cego n= 35	35 estudantes participaram do estudo; GE: redução na concentração de MP2,5 de 96,2 *μg*/m^3^ para 41,3 *μg*/m^3^.	12/13
A 03 *Effect of Portable Air Filtration Systems on Personal Exposure to Fine Particulate Matter and Blood Pressure Among Residents in a Low-Income Senior Facility: A Randomized Clinical Trial*	2018 EUA	Morishita M; Adar SD; D’Souza J; Ziemba RA; Bard RL; Spino C; Brook RD.^(20)^	Os resultados deste estudo mostraram que o uso a curto prazo de sistemas portáteis de filtragem de ar reduziu a exposição pessoal a MP2,5 e a PA sistólica entre idosos residentes em uma área urbana típica dos EUA. O uso desses sistemas relativamente baratos é potencialmente cardioprotetor contra a exposição à MP2,5.	Randomizado duplo-cego e cruzado n= 40	40 adultos não fumantes; GE: redução de 8,4 *μg*/m^3^ para 3,9 *μg*/m^3^ com o uso de filtro HEPA de baixa eficiência; Redução de 7,1 *μg*/m^3^ para 3,5 *μg*/m^3^ com o uso do filtro HEPA de alta eficiência.	12/13
A 04 *Effects of filtered fresh air ventilation on classroom indoor air and biomarkers in saliva and nasal samples: A randomized crossover intervention study in preschool children*	2019 China	Gao X; Xu Y; Cai Y; Shi J; Chen F; Lin Z; Chen T; Xia Y; Shi W; Zhao Z.^(21)^	O uso de FAVS-HEPA foi eficaz na redução de MP2,5 em ambientes internos de sala de aula. A lisozima da saliva, como um biomarcador imunológico não específico, foi significativamente inversamente associada à MP2,5 em ambientes internos. Certas bactérias nasais podem desempenhar papéis importantes na mediação da exposição à MP2,5 e nos níveis de lisozima das crianças.	Estudo cruzado randomizado n=43	43 crianças foram analisadas no estudo; GE: redução das partículas MP2,5 interno da sala de aula.	9/13
A 05 *Particulate matter concentrations in residences: An intervention study evaluating stand-alone filters and air conditioners*	2012 EUA	Batterman S; Du L; Mentz G; Mukherjee B; Parker E; Godwin C; Chin JY; O’Toole A; Robins T; Rowe Z; Lewis T.^(22)^	O estudo mostra que medições ao longo de várias estações são necessárias para caracterizar a qualidade do ar e o desempenho do filtro. A eficácia de intervenções com filtros de ar independentes depende do comportamento dos ocupantes, e estratégias para garantir o uso do filtro devem ser parte integrante das intervenções.	Ensaio clínico randomizado n=37	126 famílias com crianças com asma; GE: não houve redução da MPs2,5.	13/13
A 06 *Effectiveness of portable HEPA air cleaners on reducing indoor PM2.5 and NH3 in an agricultural cohort of children with asthma: A randomized intervention trial*	2021 EUA	Riederer, A.M.; Krenz, J.E.; TchongFrench, M.I.; Torres, E.; Perez, A.; Younglove, L.R.; Jansen, K.L.; Hardie, D.C.; Farquhar, S.A.; Sampson, P.D.; Karr, C.J.^(23)^	Neste estudo randomizado de famílias agrícolas rurais, os limpadores HEPA reduziram efetivamente os níveis de MP2,5 no quarto e na sala de estar em 60% e 42%, respectivamente, em comparação apenas à educação sobre asma, enquanto reduções significativas de amônia não foram observadas.	Ensaio clínico randomizado n=71	69 famílias com crianças com asma mal controlada; GE: redução de MP2,5 em média de 69% a 80%.	12/13
*A 07* *An indoor air filtration study in homes of elderly: cardiovascular and respiratory effects of exposure to particulate matter*	2013 UE	Karottki DG; Spilak M; Frederiksen M; Gunnarsen L; Brauner EV.^(24)^	Ao comparar a filtragem ativa e simulada do ar interno no quarto e na sala de estar por duas semanas, não encontramos melhora na manovacuometria funcional respiratória ou na função pulmonar, nem redução detectável na inflamação sistêmica, ativação de monócitos ou dano às células pulmonares nessa população idosa, incluindo pessoas em uso de medicação vasoativa, mas com níveis de exposição inicial relativamente baixos.	Randomizado duplo-cego e cruzado n=48	51 pessoas foram recrutadas; GE: redução de 30% na concentração do número de partículas na sala de estar e perto de 50% de MP2,5 na sala de estar e no quarto.	12/13

*A - artigo; E - experimental; C - controle; GE - grupo experimental; EUA - Estados Unidos da América; UE - União Europeia; MP - material particulado; PA - pressão arterial; HEPA - High Efficiency Particulate Arrestance.*

Optou-se por analisar os sete artigos que compõem a metanálise por considerarmos que esta representava a síntese mais abrangente e atualizada da literatura relevante para nossa questão de pesquisa. Ao focar nos estudos já compilados e avaliados nesta metanálise, buscamos aproveitar uma base de evidências consolidada para informar nossas análises.

A maioria dos artigos incluídos nesta RS atendeu aos critérios de avaliação da qualidade metodológica, conforme o instrumento do JBI para ensaios clínicos randomizados. Dos 47 artigos analisados, 44 (93,62%) foram considerados de boa qualidade, apresentando clareza nas respostas às questões metodológicas. No entanto, três artigos (6,38%) necessitam de informações adicionais para uma avaliação completa, pois apresentaram lacunas como: a atribuição dos grupos de tratamento não foi claramente descrita; o cegamento dos grupos de tratamento não foi evidente; não ficou claro se os avaliadores dos resultados estavam cegos quanto à atribuição do tratamento; o acompanhamento dos participantes não foi completo; as diferenças entre os grupos em termos de acompanhamento não foram adequadamente descritas e analisadas; e os resultados não foram medidos da mesma forma para os grupos de tratamento.

Apesar dessas observações pontuais, os artigos incluídos nesta RS foram considerados de boa qualidade, com a maioria dos estudos demonstrando rigor metodológico. Em relação ao risco de viés realizado com o uso da ferramenta *Cochrane Collaboration Risk of Bias*, apresentam-se os resultados na Figura 2.

**Figura 2:** Avaliação do risco de viés dos estudos incluídos na revisão sistemática segundo a ferramenta da *Cochrane Collaboration Risk of Bias,* Brasil, 2023

*Fonte: ferramenta da Cochrane Collaboration Risk of Bias^(25)^.*

Para determinar o risco de viés, utilizou-se esta ferramenta que sintetiza os resultados de estudos, afastando-se da análise isolada de cada um e seus desfechos. Sua estrutura é composta por cinco domínios, que contêm “perguntas sinalizadoras” - questões cruciais que oferecem dados complementares para avaliação. As alternativas de resposta a essas perguntas são “sim”, “provavelmente sim”, “provavelmente não”, “não”, “não há informação” e “não aplicável”. Vale ressaltar que respostas assertivas como “sim” e “não” frequentemente apontam para existência de evidências robustas. A opção “não aplicável”, por sua vez, só está disponível para perguntas de preenchimento opcional. Conforme o usuário interage com a ferramenta, as respostas são processadas por um algoritmo que, por fim, categoriza o risco de viés de cada domínio em alto, baixo ou com alguma preocupação^(25)^, conforme Figura 2.

Esta avaliação demonstrou que sete estudos representando 14,87% deles foram definidos como pouco claro; seis (12,75%) estudos apresentaram alto risco de viés; e 34 (72,38%) estudos apresentaram baixo risco de *viés.*

Na Figura 3, apresenta-se a metanálise dos artigos selecionados com o uso da ferramenta *Forest Plot*.

**Figura 3:** Gráfico *Forest Plot* para apresentação da metanálise dos estudos homogêneos utilizados nesse estudo, Brasil, 2024

*Fonte: ferramenta Floret Plot do Jamovi.*

A metanálise realizada o estudo^(26)^ traz uma significância estatística no estudo, pois mostrou um índice de evidência científica alto, em que o limite inferior é de 2.23, com Intervalo de Confiança entre 1,78 e 2,68 e limite superior de 2,68, mostrando que a evidência científica é fraca, pois os estudos são semelhantes em suas mensurações, porém a metanálise mostrou heterogeneidade dos dados.

Apesar da homogeneidade dos estudos, a metanálise revelou heterogeneidade significativa entre eles, indicando que a variabilidade não é aleatória.

## DISCUSSÃO

Nos estudos incluídos nesta RS, foi demonstrado que a filtração utilizando o filtro do tipo *High Efficiency Particulate Arrestance* (HEPA) reduz as concentrações de MP de 70% a 80% (em partículas de tamanhos variados) em aplicações de campo^(17)^.

Observa-se significância estatística no estudo, pois mostrou um índice de evidência científica alto segundo o gráfico *Forest Plot.* Foi encontrada uma diminuição modesta, mas estatisticamente significativa, na pressão arterial (PA) após a intervenção. Esta descoberta contrasta com a de vários estudos anteriores sobre filtração do ar em países com ar mais limpo, sugerindo que esses benefícios cardiovasculares possam ser mais facilmente alcançados em regiões com graves problemas de poluição atmosférica^(26)^.

A eficácia dos filtros de ar depende do tipo de poluente a ser filtrado, portanto, uma alternativa de escolha pelo filtro HEPA, que captura 99,97% das partículas presentes no ar, ou pelo filtro *Ultra Particulate Air* (ULPA), que captura 99,99% dessas partículas, representando uma alternativa segura de proteção contra riscos químicos e biológicos^(8)^.

No mecanismo de adsorção de partículas químicas e biológicas em filtros de máscaras, as partículas são atraídas para a superfície do filtro por forças eletrostáticas, forças de Van der Waals ou força hidrofóbicas^(27)^. As forças eletrostáticas são mais fortes e responsáveis pela adsorção de partículas carregadas positivamente ou negativamente^(27)^. As máscaras geralmente são feitas de materiais que possuem carga elétrica negativa, o que ajuda a atrair partículas carregadas positivamente, como poeira, pólen e vírus, cada uma com características e finalidades específicas, muitas delas utilizando propriedades eletrostáticas para aumentar a eficiência da filtragem, em que as mais comuns e utilizadas pelos trabalhadores da saúde são as máscaras cirúrgicas que filtram o ar exalado pelo usuário para proteger o ambiente e, em menor grau, filtram o ar inalado de gotículas maiores^(27)^.

N95 ou PFF2, que são respiradores de peça semifacial descartáveis que se ajustam firmemente ao rosto, filtram pelo menos 95% das partículas aero dispersas, incluindo as de 0,3*µ m*. Possuem múltiplas camadas, muitas das quais com tratamento eletrostático para atrair partículas. PFF3, a mais alta classe de filtros de partículas descartáveis, com eficiência mínima de 99,95% das partículas de 0,6 *µm*, protege os trabalhadores em ambientes com substâncias altamente tóxicas, manuseio de materiais radioativos e áreas de alto risco biológico^(28)^.

As forças hidrofóbicas são forças que repelem a água, e as máscaras geralmente são feitas de materiais que são hidrofóbicos, o que ajuda a atrair partículas hidrofílicas, como vapor d’água e gotículas. O tamanho das partículas também afeta sua adsorção em filtros de máscaras, em que as partículas maiores são mais facilmente atraídas para a superfície do filtro do que as partículas menores. Isso ocorre porque as partículas maiores têm mais superfície para interagir com as forças de adsorção^(29)^.

A eficiência de um filtro de máscara na adsorção de partículas químicas e biológicas depende de vários fatores, incluindo o tipo de material do filtro, as propriedades da superfície do filtro, o tamanho das partículas e a concentração das partículas^(30)^.

As máscaras são feitas usando a técnica de sopro por fusão, um processo no qual cargas elétricas são injetadas no material, criando um campo elétrico quase permanente que fornece filtragem suficiente de MP por meio de uma força eletrostática. Substâncias fibrosas não tecidas, como polipropileno, polietileno, ácido polilático e politetrafluoroetileno (PTFE), são os materiais mais estudados^(30)^.

As fibras não tecidas de polipropileno fundido por sopro têm propriedades estáveis, um diâmetro de fibra fina (0,5-4 *μm*), alta porosidade, boa permeabilidade ao ar e boa resistência à filtragem. Portanto, o desempenho do material de filtro preparado com polipropileno é muito melhor do que o de outros materiais^(27)^.

Estudo randomizado, duplo-cego, de intervenção cruzada de três vias, com filtração utilizando o filtro do tipo HEPA de baixa eficiência (LE) e filtro HEPA de alta eficiência (HE) do ar ambiente usando sistemas de purificação de ar no quarto e na sala principal das residências, evidenciou que o filtro LE remove 99% das partículas com tamanho de 2,0 *μm*, enquanto o filtro HE remove 99,97% das partículas com tamanho de 0,3 *μm*, complementando que esta abordagem prática e relativamente barata pode ser uma ferramenta eficaz para reduzir os efeitos à saúde relacionados às MP2,5^(20)^.

O filtro HEPA é um sistema de filtragem de ar amplamente utilizado em ambientes onde a qualidade do ar é crítica. Ele é capaz de reter uma grande variedade de partículas, incluindo as pequenas, o que o torna fundamental em diversas aplicações. Eles operam através de três mecanismos principais para reter partículas: interceptação: partículas que passam muito perto de uma fibra do filtro são fisicamente aprisionadas e aderem à fibra; impacto inercial: partículas maiores não conseguem desviar das fibras do filtro e colidem com elas devido à sua massa, ficando presas; difusão (movimento browniano): partículas ultrafinas (menores que 0,1 *µm*) realizam um movimento aleatório errático (movimento browniano) devido às colisões com moléculas de gás. Esse movimento aumenta a probabilidade de colisão com as fibras do filtro e captura^(31)^.

O uso de filtros HEPA em ambientes hospitalares é crucial para reduzir a incidência de infecções hospitalares causadas por bactérias, vírus e fungos aerotransportados. Eles removem patógenos que causam doenças como tuberculose, influenza e outras infecções respiratórias, contribuindo para a segurança de pacientes e profissionais de saúde. Sendo assim, a utilização de filtros HEPA contribui para uma melhor qualidade do ar interior, eliminando partículas finas que podem ser inaladas e causar problemas de saúde a longo prazo^(32)^.

As evidências demostram também que o uso de máscaras faciais do tipo N-95 por duas horas durante o pico de tráfego resulta em reduções no óxido nítrico exalado, múltiplas citocinas inflamatórias no condensado do ar exalado, mas nenhum efeito benéfico claro na função endotelial ou sistêmica, com evidências baixas de que o uso deste tipo de EPI reduz a inflamação sistêmica ou o estresse oxidativo, evidenciando-se aqui que são máscaras seladas e bem ajustadas^(33)^.

A classificação N-95 é designada pela eficiência de filtragem mínima de 95%, composta por várias camadas de tecido não tecido de polipropileno. A eficiência deste modelo é observada pela capacidade de filtração, ocasionada pela carga eletrostática proporcionada pela fibra de polipropileno, cuja ação é fundamental para garantir a proteção de quem a utiliza^(28)^.

Estudo examinou o impacto da filtragem do ar interno em mudanças agudas nas medidas clínicas de saúde respiratória e cardiovascular em uma comunidade Primeiras Nações. Esse primeiro estudo cruzado foi realizado em uma reserva das Primeiras Nações em Manitoba, Canadá, incluindo 37 residentes em 20 casas. Estudo que examinou a qualidade do ar em Primeiras Nações em Manitoba (Canadá), com um filtro de ar eletrostático *3M Filtrete FAP03-RS Ultra Clean Air Purifier* e um filtro placebo, verificou que houve melhora nas medidas da função pulmonar, PA e função endotelial^(18)^.

Outro estudo, com o uso de respiradores com filtro modelo FAP04, *3M Filtrete* de partículas, pode produzir benefícios cardiovasculares, melhorando a função nervosa autônoma e reduzindo a PA. O estudo foi conduzido no *campus* Fenglin da Universidade Fudan, localizado na área urbana central de Xangai. Esta intervenção foi concebida como um estudo randomizado, duplo-cego e cruzado^(34)^.

Os dez quartos foram randomizados em dois grupos de cinco quartos cada. Um grupo usou um purificador de ar colocado no centro do quarto por 48 horas, o que correspondeu a dois finais de semana, seguido por um período de *washout* de duas semanas, e então usou um purificador de ar falso sob as mesmas condições por mais 48 horas. O outro grupo simplesmente inverteu a ordem em que os purificadores de ar reais e falsos foram usados. Todos os quartos usaram os mesmos purificadores de ar qualificados (modelo FAP04, *3M Filtrete*, Xangai, China), com a única diferença sendo a remoção da tela filtrante nos purificadores falsos. O recurso de detecção automática de poluição do ar dos purificadores de ar foi desativado em ambos os grupos. Todos os participantes e a equipe de pesquisa estavam cegos para a designação dos grupos^(34)^.

Estudo prospectivo cruzado randomizado e não cego de 22 voluntários asiáticos saudáveis (nove homens e 13 mulheres) comparou a proporção de contagens de partículas dentro e fora de duas máscaras de oxigênio: a *FiltaMask* e uma máscara do tipo Hudson (*Well Lead Medical*, Guangzhou, China). Os resultados demonstraram que a *FiltaMask* reduziu a concentração de partículas de 0,02 *μm* a 12 *μm* dentro da máscara em relação à concentração ambiente. O grau de redução foi significativamente maior do que o observado com uma máscara tipo Hudson, indicando que a redução não se deu simplesmente à diluição da concentração de partículas pelo oxigênio^(35)^.

Foram recrutadas 43 crianças de 5 a 14 anos de Songjiang, China, um subúrbio de Xangai, para participar de estudo duplo-cego, randomizado e cruzado. Amostras de MP2,5 integradas no tempo de 48 horas foram coletadas em filtros de PTFE (Teflon) de 37 mm usando monitores ambientais pessoais (PEMs; SKC, EUA) com bombas AirChek XR5000 (SKC, EUA). Foi descoberto que o filtro de ar interno reduziu o potencial oxidativo medido da exposição interna e pessoal à MP. Utilizando contribuições de fontes do modelo EPA MPF 5.0, foram identificadas as fontes de elementos em MP2,5 que se infiltraram no ambiente interno, sendo reduzidos em ambientes fechados por um filtro de ar. Foi descoberto que o filtro de ar interior reduziu o potencial oxidativo medido da exposição pessoal às MPs^(36)^.

Embora as propriedades de aerossol dos bioaerossóis sejam geralmente consideradas semelhantes às dos não bioaerossóis, a seleção e manutenção do respirador são complicadas pela natureza biológica. Os bioaerossóis incluem bactérias, vírus, fungos, algas e ácaros. Além disso, produtos biológicos como pólen, endotoxinas, proteínas e excrementos animais formam aerossóis. Tanto as formas viáveis quanto as não viáveis de bioaerossóis podem ser perigosas para a saúde. Os bioaerossóis infecciosos produzem efeitos adversos à saúde devido à sua capacidade de incubar, crescer, multiplicar-se e produzir substâncias tóxicas^(37)^.

### Limitações do estudo

O estudo traz evidências preliminares sobre a eficácia do filtro HEPA na remoção de material particulado. No entanto, são necessárias mais pesquisas para confirmar e expandir esses resultados, considerando a complexidade do tema e a diversidade de fatores que podem influenciar a qualidade do ar.

### Contribuições para a área da enfermagem

Esta pesquisa avaliou a efetividade de diferentes filtros na remoção de material particulado de diversos tamanhos em ambientes de cuidados simulados e não simulados. Adicionalmente, os resultados obtidos serão utilizados para o desenvolvimento de um novo filtro para máscara HeLP, uma máscara ergonômica e sustentável feita com material passível de desinfeção, o qual passará por rigorosos testes de proteção contra riscos químicos e biológicos relevantes para trabalhadores da área da saúde. Serão fornecidos dados essenciais para selecionar filtros ideais, otimizar protocolos de manutenção e, crucialmente, embasar o desenvolvimento de tecnologias de proteção individual mais eficazes para os trabalhadores da área da saúde.

Essa máscara irá proteger os trabalhadores da área da saúde contra algumas doenças, como rinite alérgica, hipotireoidismo, enxaqueca, asma, hipertensão arterial, sistêmica, fibromialgia e até mesmo de neoplasias malignas.

Nesse sentido, a NR32, que aborda proteção contra os riscos ocupacionais químicos aos quais os trabalhadores da área da saúde estão sujeitos, torna-se relevante. A criação e implementação de políticas institucionais e regulamentações adequadas são imprescindíveis para proteção dos trabalhadores da área da saúde.

## CONCLUSÕES

Os resultados deste estudo indicam que a filtração com filtro HEPA foi superior na redução de MP no ar ambiente (99,97% de captura). Embora o filtro ULPA apresente uma captura ligeiramente maior (99,99%), ambos representam alternativas seguras contra riscos químicos e biológicos.

Outros filtros também foram testados além do HEPA, como os filtros de PTFE (Teflon) de 37 mm, apresentando uma redução no potencial oxidativo da exposição interna ao MP2.5. A *FiltaMask N-95* e o tipo Hudson reduziram partículas de 0,02 µm a 1 µm dentro da máscara. E as máscaras *3M Filtrete FAP03-RS Ultra Clean Air Purifier* reduziram os níveis internos de MP, aliviando os efeitos da poluição.

## DISPONIBILIDADE DE DADOS E MATERIAL

Os dados de pesquisa estão disponíveis no corpo do artigo.
